# Resilience and positive coping style affect the relationship between maladaptive perfectionism and academic procrastination among Chinese undergraduate nursing students

**DOI:** 10.3389/fpsyg.2022.1014951

**Published:** 2022-10-20

**Authors:** Haitao Huang, Yueming Ding, Yiming Zhang, Qianwen Peng, Yipei Liang, Xiao Wan, Chaoran Chen

**Affiliations:** ^1^School of Nursing and Health, Institute of Nursing and Health, Henan University, Kaifeng, China; ^2^School of Business, Institute of Business Administration, Henan University, Kaifeng, China

**Keywords:** resilience, coping style, maladaptive perfectionism, academic procrastination, undergraduate nursing students

## Abstract

**Background:**

Previous studies have not investigated the role of resilience and coping style on the association between maladaptive perfectionism and academic procrastination among nursing undergraduates. However, how to mobilize the learning enthusiasm of nursing students and reduce the incidence of academic procrastination is an important factor to reduce nursing loss and improve nursing quality.

**Objectives:**

To investigate the influence of maladaptive perfectionism, resilience and coping style on academic procrastination among Chinese undergraduate nursing students.

**Methods:**

A cross-sectional study was conducted. A convenience sampling method was used to select 665 nursing undergraduates from March to May 2022 in China. Maladaptive perfectionism, coping style, resilience, and academic procrastination were measured using questionnaires. The descriptive analysis, Pearson’s correlation analysis and the Hayes’ PROCESS Macro in SPSS 25.0 were used to test the model.

**Results:**

The results showed that nursing undergraduates’ maladaptive perfectionism, resilience, positive coping style and academic procrastination were significantly correlated between every two variables, with coefficients ranging between −0.290 and 0.584. In addition, resilience played a partial mediating role in maladaptive perfectionism and academic procrastination, accounting for 15.70% of the total effect; in the meantime, this process was moderated by positive coping style.

**Conclusion:**

Maladaptive perfectionism positively predicted nursing undergraduates’ academic procrastination; as a mediating mechanism with moderating, resilience and positive coping style further explained how maladaptive perfectionism promoted the academic procrastination of nursing undergraduates. Understanding this mechanism is of great significance for nursing educators to reduce the risk of academic procrastination in nursing undergraduates.

## Introduction

Due to growing demand for nursing, unbalanced nurse-to-patient ratio and increasing job pressures, it is becoming increasingly difficult to recruit and retain nurses globally, which is undermining nursing outcomes worldwide (Jarrar et al., 2018; Li et al., 2020). Therefore, there is an urgent need for us to train more nursing students. However, many nursing students do not consider nursing an interesting major for various reasons (Larsen et al., 2012; Sela-Vilensky et al., 2020). This leads to higher dropout rates and prevents us from producing more professional and enthusiastic nurses (Bakker et al., 2019). In this case, reducing the incidence of academic procrastination (AP) is considered to be one of the effective ways to reduce nursing student attrition (Guo et al., 2019). Procrastination refers to the non-adaptive behavior that people involuntarily postpone a predetermined plan without a clear reason (Kandemir, 2014). AP is a form of procrastination in school situations and is related to the fulfilment of studying tasks. Some researchers interpreted AP as emotional discomfort experienced by individuals who delay embarking on a task that must eventually be completed (Lay and Schouwenburg, 1993). AP is common among medical college students, with about 13.8 to 49.9% of medical students reporting procrastination on learning tasks (Madhan et al., 2012; Mortazavi et al., 2015). AP will not only lead to a decline in school achievement, but also have a negative impact on college students’ learning attitude (Kim and Seo, 2015; Balkis and Duru, 2016). In addition, college students with AP are at higher risk of negative emotions such as depression and anxiety (Onwuegbuzie, 2004; Martinčeková and Enright, 2020). what is worse, it even has a higher risk of taking their own life (Klibert et al., 2011; Flett et al., 2016). As a result, reducing the incidence of AP among nursing students is essential for consolidating the nursing force. Although there have been previous international studies on the AP of nursing students, few of these have involved Chinese nursing undergraduates (Custer, 2018; Guo et al., 2019). In China, nursing undergraduates are playing an increasingly important role in nursing education (Fu et al., 2022). Data from China show that nurses with advanced diplomas or bachelor’s degrees are the most needed workforce at all levels of health care and in the primary care sector (Wang et al., 2016; Fu et al., 2022). The undergraduate stage is the key stage for the formation of professional concept, value and professional ability of nursing students (Gao et al., 2022). In view of the above considerations, it is essential to investigate the risk factors and the mechanisms associated with AP among Chinese nursing undergraduates, which may offer significative guidance for future education.

### Background

Cognition and Behavior Theory (CBT) indicates that individual behavior is largely influenced by cognition, and studies have shown that procrastination is closely related to irrational worry and self-criticism (Knaus, 1973). Procrastinators often feel less confident in their ability to complete tasks, and thus delay the start of tasks (Knaus, 1973). Perfectionism is a tendency to pursue perfection in everything, and it is a personality trait that has an important influence on people’s emotions and behaviors (Frost et al., 1990; Pishghadam and Akhondpoor, 2011). Current research finds that perfectionism is a multidimensional structure, which has both positive aspects, called adaptive perfectionism and negative aspects, called maladaptive perfectionism (MP; Egan et al., 2011). MP refers to the tendency of critical self-evaluation and worry about others’ expectations and comments (Frost et al., 1990). Previous studies have shown that the proportion of perfectionists among nursing students in China is high, and more than one half of nursing students can be classified as perfectionists (Cheng and Liu, 2017). Studies have indicated that MP is associated with eating disorders, obsessive–compulsive disorders, depression, learner anxiety and suicidal ideation (Pishghadam and Akhondpoor, 2011; Kiamanesh et al., 2015; Jing and Jianing, 2018; Dorevitch et al., 2020). Research on the association between MP and procrastination has found that MP was significantly predictive of procrastination. Ferrari’s study of both procrastinators and non-procrastinators found that procrastinators reported more MP (Ferrari et al., 1992). Later, Frost et al. (1993) investigated the association between perfectionism and procrastination. They found that concern over mistakes was significantly correlated with attitude toward procrastination and parental expectations and criticism were significantly correlated with frequency of procrastination (Frost et al., 1993). Previous studies have documented a link between MP and procrastination among college students (Chi et al., 2012; Chen et al., 2013). However, results on the extent to which MP affect the AP of nursing undergraduates in mainland China are still lacking. Given that nursing undergraduates are an important part of the future nursing force in China, it is essential to investigate the association between MP and AP.

Resilience was defined as “an individual’s behavioral tendency to adapt to changing circumstances and the ability to recover from stressful situations” (Block and Kremen, 1996). According to the Resiliency Model proposed by Richardson, resilience is not only affected by the individual’s cognition and behavior, but also has a protective effect on the cognition and behavior (Richardson, 2002). Previous research has shown that as a special personality trait, there is a certain relationship between MP and self-esteem (Chen et al., 2013), while self-esteem and resilience have a certain correlation (Chung et al., 2021). Therefore, we speculate that there is also a certain relationship between MP and resilience. In addition, the essence of AP is the fear of failure and the lack of self-regulation ability in the face of the pressure of academic tasks (Chen et al., 2013). As a positive psychological trait, resilience can mobilize individual protective resources, help individuals cope well with adverse situations and maintain positive emotional states (Klika and Herrenkohl, 2013), so it should play a positive role in reducing AP.

Coping is an individual’s cognitive and behavioral effort to reduce the negative effects of stress, which is usually divided into positive coping style (PCS) and negative coping style(NCS)(Patterson and McCubbin, 1987). Among them, the PCS refers to the individual’s problem-solving oriented, actively seeking internal and external resources, so as to construct problem-solving strategies; The NCS refers to that individuals pay more attention to their own emotional experience when facing problems and solve problems by denying, escaping and fantasizing (Patterson and McCubbin, 1987). Although resilience may have a positive effect on AP and in general, it does not affect all individuals equally. In other words, individuals with different levels of coping style may differ in the patterns of correlation among resilience and AP. According to the Stress-Diathesis Model, individual coping style has an important influence on psychology and behavior (Monroe and Simons, 1991). Researchers have found that in positive situations, protective coping styles enhance the positive psychological and behavioral effects, while NCS weaken the positive psychological and behavioral effects(Hollister-Wagner et al., 2001; Fergus and Zimmerman, 2005). In conclusion, it can be concluded that PCS will enhance the positive effect of resilience on AP to some extent, while NCS will weaken the protective effect of resilience on AP.

### The present study

**Figure 1:**
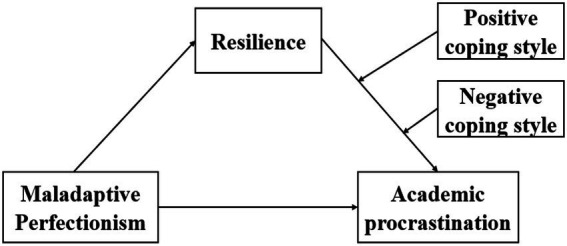
The theoretical model of this study.

Based on Cognitive Behavioral Theory, Resiliency Model and Stress-Diathesis Model, this study examined the effects of MP, resilience and coping style on AP among undergraduate nursing students in mainland China. The following hypotheses have been proposed (Figure 1):

*H1*: Maladaptive perfectionism is positively correlated with academic procrastination in undergraduate nursing students;

*H2*: Maladaptive perfectionism is negatively related to resilience in undergraduate nursing students;

*H3*: Resilience is negatively related to undergraduate nursing students’ academic procrastination

*H4*: Resilience partially mediates the association between maladaptive perfectionism and academic procrastination

*H5*: Positive coping style plays a moderating role in the association between resilience and academic procrastination.

*H6*: Negative coping style plays a moderating role in the association between resilience and academic procrastination.

## Materials and methods

### Design

A cross-sectional survey was conducted from March to May 2022.

### Participants

A convenience sampling method was used to recruit nursing undergraduates from two undergraduate universities in Henan, China from March to May 2022. Participants meet the following inclusion criteria: (1) full-time nursing undergraduates in Grade1, Grades2 and Grade3; (2) Know the purpose of the research and volunteer to participate in the research; And the exclusion criteria were students who did not complete all questionnaires for various reasons. Equation *N* = 4Uα^2^S^2^/δ^2^ (Ni et al., 2009) was used to calculate the sample size. *S* = 0.59 is calculated from the pre-survey, the allowable errorδis set to 0.1, and *α* is set to 0.05, so *N* = 4 × 1.96^2^ × 0.59^2^/0.1^2^ = 535. Taking into account the sampling error and possibility of invalid questionnaires, we distributed a total of 700 questionnaires. Finally, after removing 35 unqualified questionnaires, a total of 665 valid questionnaires were obtained.

### Data collection

Before sampling, we discussed the contents and procedures of the questionnaire with the competent authority involved in each university. After obtaining permission from the competent authority, investigators will begin handing out paper questionnaires to students as they gather in a classroom (about 50 students at a time). Participants were not given any incentive or inducement throughout the test. Furthermore, participants were told that their answers to the questionnaire would be anonymous and confidential, and that the data collected would only be used for academic study.

### Ethical considerations

This study has been reviewed and approved by Institutional Review Board of Henan Provincial Key Laboratory of Psychology and Behavior (reference: 20220107001), and is carried out in accordance with the Declaration of Helsinki.

### Instruments

#### Demographic information

A demographic questionnaire assessed participant characteristics including age, gender, and residential area, etc.

#### The Chinese frost multidimensional perfectionism scale (CFMPS)

Frost Multidimensional Perfectionism Scale was compiled by Frost in 1990 to measure the cognitive, emotional and behavioral performance of perfectionists (Frost et al., 1990). Zi and Zhou (Fei and Xu, 2006) modified it according to the Chinese culture in 2006, and conducted reliability and validity tests to form the Chinese version of FMPS. The questionnaire included five dimensions of “Concern over Mistakes (CM),” “Organization,” “Personal Standard (PS),” “Parental Expectations (PE)” and “Doubts about Actions (DA),” with a total of 27 items. “Organization” and “PS” was considered as adaptive perfectionism. “CM,” “PE” and “DA” were considered maladaptive perfectionism. Cronbach’s α of the five dimensions were 0.76, 0.81, 0.74, 0.70 and 0.64, respectively. In this study, 5-level Likert scale was used, with 1 indicating that “inconsistent” and 5 indicating that it was “completely consistent.” Confirmatory factor analysis (CFA) was conducted to determine the convenience of the scale structure with the data collected within the scope of this study. CFA found that scale factor structure had an acceptable fit with the data (*χ*^2^/*df* = 2.513，RMSEA = 0.045, GFI = 0.929，AGFI = 0.915，IFI = 0.981，CFI = 0.979，TLI = 0.925). The internal consistency coefficient of maladaptive perfectionism in current study was 0.80.

#### Simplified coping style questionnaire (SCSQ)

The SCSQ was developed by Xie according to the characteristics of Chinese people (Xie, 1998). It was composed of two dimensions of positive coping and negative coping, with a total of 20 items. 4-level Likert scale were used in the questionnaire (1 = never, 4 = always). Positive coping consists of 12 items, which mainly describes some characteristics of positive coping, such as “try to see the good side of things”; Negative coping consists of 8 items, focusing on the characteristics of negative coping, such as “imagining that some kind of miracle might happen to change the status quo.” The SCSQ had good validity in current study (CFA: χ^2^/*df* = 2.727, RMSEA = 0.048, GFI = 0.931，AGFI = 0.925，IFI = 0.976，CFI = 0.923，TLI = 0.953). The Cronbach’s α of the two subscales in this study were 0.80 and 0.74, respectively.

#### Connor-Davidson resilience scale (CD-RISC)

CD-RISC was developed by Connor and Davidson in 2003, which includes 25 items and is divided into five dimensions: tenacity, tolerance of negative effect, positive acceptance of change, control and spiritual influences (Connor and Davidson, 2003). The Chinese version of CD-RISC was revised by Yu retaining 25 items of the original scale and adjusting it to three dimensions: tenacity, strength and optimization; Likert 5 scores were used (0 = never, 4 = almost always). CD-RISC (Chinese version) has been shown to have good reliability in the Chinese population (Cronbach’s *α* = 0.91; Yu and Zhang, 2007). The CD-RISC had good validity and reliability in current study (CFA: χ^2^/*df* = 2.645，RMSEA = 0.034, GFI = 0.936，AGFI = 0.947，IFI = 0.923，CFI = 0.958，TLI = 0.939).The Cronbach’s α was 0.91.

#### Aitken procrastination inventory (API)

API is a self-assessment scale developed by Aitken (1982) to evaluate the persistent academic procrastination of college students. It has a total of 19 items and has been proved to have good internal consistency in the Chinese context (Chen, 2008). Likert 5 scores were used, with “1” meaning “completely inconsistent” and “5” meaning “completely consistent.” Items 2, 4, 7, 11, 12, 14, 16, 17, 18 were inversely scored. Sample items are “I always start solving problems at the last minute; A high score indicated a higher degree of academic procrastination. The Cronbach’s *α* in our study were 0.82.

### Statistical analyses

All the data were analyzed using IBM SPSS statistics 25.0 and the PROCESS macro3.3. The demographic characteristics of the participants were represented by descriptive statistics. Pearson correlation analysis was used to explore the association between MP, PCS, NCS, resilience and AP. Harman’s single-factor test was used to evaluate the common method bias derived from self-reported data (Podsakoff et al., 2003). The mediating effect of resilience between MP and AP was tested by PROCESS Model 4 (Hayes, 2017). The moderating role of PCS and NCS was examined by the Model 16. In addition, we used the 5,000 resample bootstrapping method with a 95% CI to test the effect of the independent variable on the dependent variable through the mediating variable. All *p* values are two-sided, with *p* < 0.05 indicating a statistically significant result. The report of this study is strictly in accordance with the STROBE Statement (von Elm et al., 2007).

### Validity and reliability/rigour

Firstly, all the instruments used in study have been adjusted and verified by Chinese culture and have good validity and reliability. In addition, before the formal investigation, all investigators were trained on registration, checking the completeness of questionnaires, and training on ethical tenets of conducting research. To reduce the risk of self-reported bias, the identities of all participants are kept strictly confidential. Finally, to ensure the rigor and accuracy of the statistical analysis, we invited a statistics professor to examine the data processing.

## Results

### Common method biases tests

Harman’s single-factor test extracted 20 factors with eigenvalues greater than 1. The first factor explained 14.967% of the total variances, which is below the recommended threshold of 40% (Podsakoff et al., 2003). It suggests that common method bias is unlikely to confuse the interpretation of data analysis results.

### Participants’ characteristics

A total of 665 participants completed the final survey, with an average age of 19.86 ± 1.19 years. Table 1 shows the basic demographic characteristics of participants.

**Table 1:** Demographic characteristics of the study participants (*N* = 665).

Variables		Number	%
*Gender*			
	Male	149	22.41
	Female	516	77.59
*Grade*			
	Freshman	275	41.35
	Sophomore	233	35.04
	Junior	157	23.61
*Residential area*			
	Urban	421	63.31
	Rural	244	36.69
*Whether or not the only child*			
	Yes	93	13.98
	No	572	86.02
*Monthly income (¥)*			
	< 3,000	242	36.39
	3,000–6,000	338	50.83
	> 6,000	85	12.78

### Descriptive analysis and correlations between overall variables

Means, standard deviations (SD), and Pearson correlations of each variable were shown in Table 2. The average score of MP was (2.893 ± 0.591), resilience was (2.775 ± 0.494), AP was (2.549 ± 0.604), PCS was (2.547 ± 0.433) and NCS was (2.720 ± 0.546). As can be seen from the range of item scores, the scores of the five variables were basically at the medium level.

**Table 2:** Descriptive statistics and correlations (*N* = 665).

Variables	1	2	3	4	5	6	7	8	9	10	11	Range	M ± SD
1.MP	1											1–5	2.893 ± 0.591
2.CM	0.854**	1										1–5	2.386 ± 0.856
3.PE	0.669**	0.311**	1									1–5	3.214 ± 0.720
4.DA	0.692**	0.436**	0.0745**	1								1-5	3.258 ± 0.751
5.Resilience	−0.105**	−0.165**	−0.090*	−0.137**	1							0-4	2.775 ± 0.494
6.Tenacity	−0.096*	−0.150**	−0.099*	−0.147**	0.950**	1						0-4	3.374 ± 0.557
7.Strength	−0.103**	−0.162**	−0.062*	−0.101**	0.901**	0.756**	1					0-4	3.600 ± 0.535
8.Optimization	−0.072	−0.115**	−0.159**	−0.086*	0.725**	0.564**	0.617**	1				0-4	3.368 ± 0.560
9.AP	0.215**	0.223**	0.088*	0.181**	−0.290**	−0.262**	−0.316**	−0.149**	1			1-5	2.549 ± 0.604
10.PCS	−0.083*	−0.112**	−0.079*	−0.090*	0.584**	0.528**	0.560**	0.443**	−0.261**	1		1-4	2.547 ± 0.433
11.NCS	0.181**	0.186**	0.065	0.136**	−0.057	−0.063	−0.095*	0.072	0.286**	0.037	1	1-4	2.720 ± 0.546

MP, Maladaptive perfectionism; CM, Concern over Mistakes; PE, Parental Expectations; DA, Doubts about Actions; AP, Academic procrastination; PCS, Positive coping style; NCS, Negative coping style.

* *p* < 0.05;

** *p* < 0.01.

MP was significantly correlated with resilience (*r* = −0.105, *p* < 0.01), AP (*r* = 0.215, *p* < 0.01), PCS (*r* = −0.083, *p* < 0.05) and NCS (*r* = 0.181, *p* < 0.01). Moreover, resilience was significantly correlated with AP (*r* = −0.290, *p* < 0.01) and PCS (*r* = 0.584, *p* < 0.01). Finally, PCS and NCS are significantly correlated with AP, respectively, (PCS: *r* = −0.261, *p* < 0.01; NPS: *r* = 0.286, *p* < 0.01).

### Mediating effect with moderating analysis

In the first place, multiple linear regression analysis showed that gender and age had a significant influence on AP. As a result, they were included as covariates in the moderated mediation analysis.

**Figure 2:**
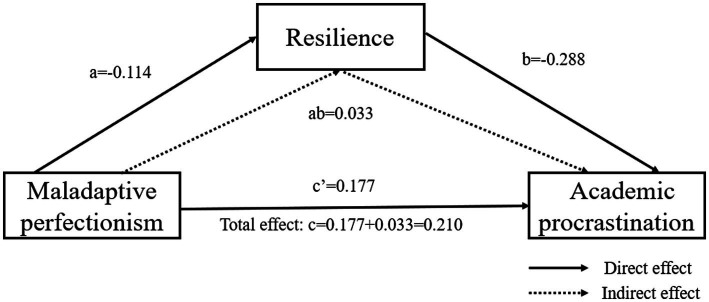
Mediating effect of resilience.

In the second place, PROCESS Macro model 4 was used to analyze the mediating role of resilience. MP significantly positively predicts AP after controlling for age and gender (*c* = 0.210, *t* = 5.492, *p* < 0.001). The H1 is verified. When MP and resilience entered the regression equation together, the predictive effect of MP on AP is still significant (*c*′ = 0.177, *t* = 4.799, *p* < 0.001). MP has a significant negative predictive effect on resilience (*a* = −0.114, *t* = −2.984, *p* < 0.001), and resilience has a significant negative predictive effect on AP (*b* = −0.288, *t* = −7.441, *p* < 0.001). This manifested that resilience partially mediates the relationship between MP and AP. The H2 and H3 are supported. Bootstrap method test with percentile bias correction indicated that resilience has a significant mediating effect between MP and AP, ab = 0.033, Boot SE = 0.013 and 95%CI = (0.008, 0.060). The contribution rates of indirect effects in the total effect was ab/(ab + c′’) = (0.033/0.210) = 15.70%. In other words, resilience is the mediating effect of 15.70% in the association between MP and AP (The H4 was supported). Figure 2 shows the direct, indirect, and total effects.

Finally, PROCESS Macro model 16 was used to analyze the moderating role of PCS and NCS. The predictive variables in the regression model are standardized, and age and gender are taken as control variables. If the model meets the following three conditions, there will be a mediating mechanism with moderating effect: (a) In Model 1, MP has a significant overall impact on AP; (b) in Model 2, MP is significantly negatively associated with resilience; (c) in Model 3, resilience has a significant main effect on AP, and the interaction term between resilience and PCS/NCS has a significant effect on AP. The results showed that the analysis of the moderating effect of PCS fully met the above conditions, that is to say, PCS moderated the association between resilience and AP, while the moderating role of NCS were not significant, which supports H5 and rejects H6.

**Figure 3:**
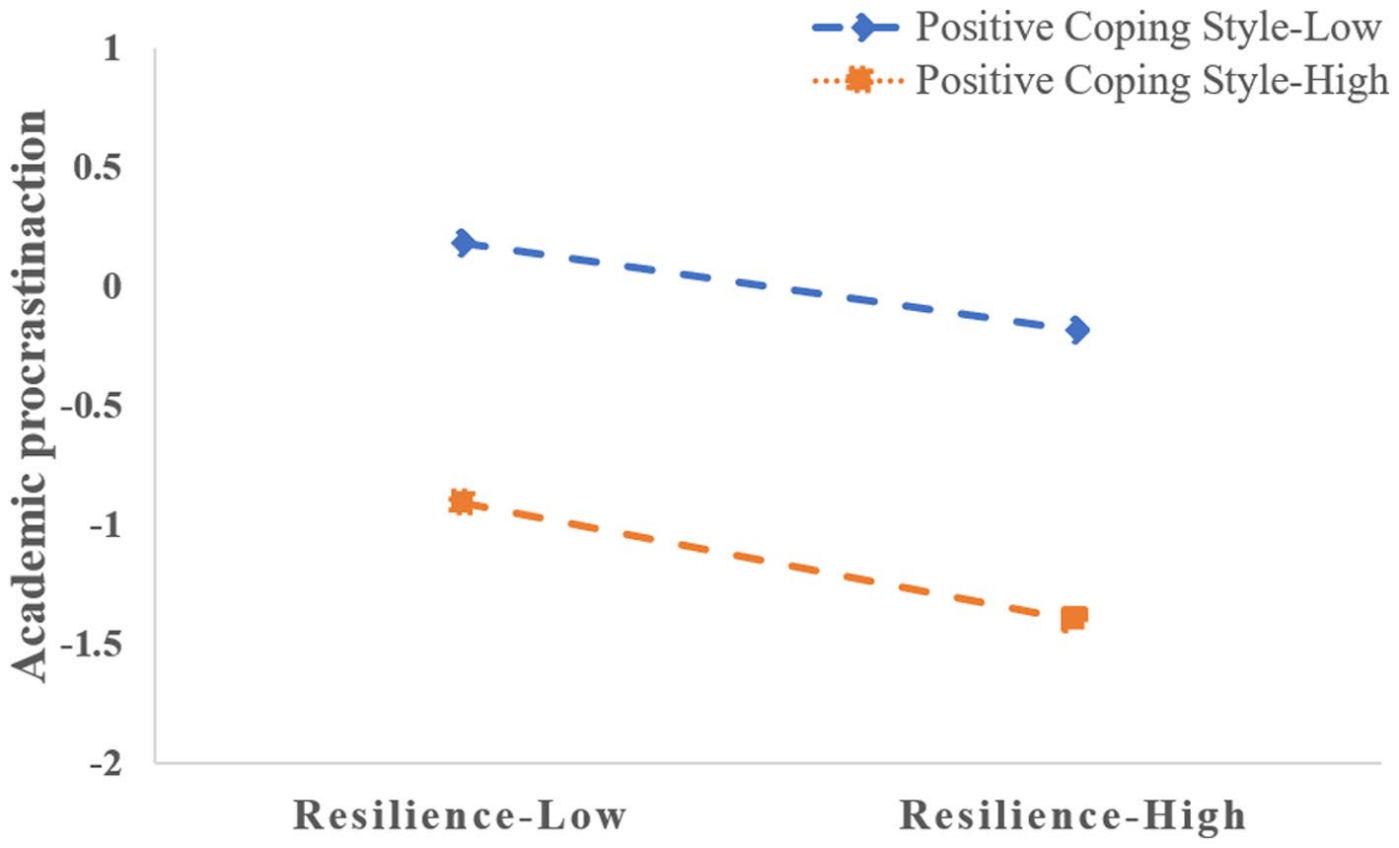
Moderating effect of positive coping style.

Simple slope analysis was used to further visually investigated the moderating role of PCS. Whether PCS is at a high or low level, resilience had a significant effect on AP. The difference was that for nursing undergraduates with high PCS, the effect of resilience on AP showed an obvious trend of strengthening (*β* = −0.247, *t* = −4.589, *p* < 0.001); However, for nursing undergraduates with low PCS, the effect of resilience on AP was still significant but decreased (*β* = −0.115, *t* = −2.104, *p* < 0.05). Figure 3 has shown the moderating role of PCS between resilience and AP (Table 3).

**Table 3:** The model of mediating effect with moderating.

Predictive variable	Model 1(criterion: AP)		Model 2(criterion: Resilience)		Model 3(criterion: AP)	
	*β*	*t*	*β*	*t*	*β*	*t*
Gender	0.100	1.982*	−0.113	−1.818	0.146	2.069*
Age	−0.110	−2.459**	0.101	1.943	−0.096	−1.135*
MP	0.210	5.491**	−0.114	−2.984*	0.134	3.763**
Resilience					−0.181	−4.008**
PCS					−0.151	0.001**
NCS					0.247	6.822**
Resilience × PCS					−0.066	−2.185*
Resilience × NCS					0.046	1.427
*R* ^2^	0.051		0.045		0.213	
*F*	5.934**		5.214**		16.023**	

MP, Maladaptive perfectionism; AP, Academic procrastination; PCS, Positive coping style; NCS, Negative coping style.

* *p* < 0.05;

** *p* < 0.001.

## Discussion

Our study aimed to explore the association between MP, resilience, PCS, NCS and AP among Chinese nursing undergraduates. First, results show that except for NCS, there was a significant correlation between each of the two variables among the remaining variables. Second, MP has a positive effect on AP, and resilience plays a partial mediating role in the relationship. In the end, our study confirms that PCS play a moderating effect in the mediating mechanism. To be specific, the resilience of nursing students with a high level of PCS has a higher predictive effect on AP than that of nursing students with a low level of PCS.

In this study, the MP of undergraduate nursing students were slightly higher than the results of Zheng (2011), which may be related to the fact that the participants of the latter were ordinary college students, while this study was nursing students. Due to the particularity of the nursing profession and the requirements of the future occupational environment, accurate performance is an underlying principle of nursing education (Si and Lee, 2022). Nursing students are usually expected to perform all tasks perfectly. Furthermore, since every nursing practice is closely related to the quality of nursing, nursing students may experience high levels of stress due to an excessive fear of making mistakes, leading to highly MP. The resilience is at a medium level, which is consistent with the precious result (Wang, 2016). indicating that the participants have certain positive psychological resources, but still need to be strengthened. In addition, the score on AP was lower than in previous study (Cho and Lee, 2022), a possible reason being that the majority of respondents in this study were female student. And studies have shown that female are less likely than male to procrastinate on academic tasks for fear of lower academic performance (Lu et al., 2021). Finally, compared to previous study (Cheng et al., 2021), PCS slightly scored lower and NCS scored higher, possibly because during the COVID-19 pandemic, fear of infection and death may predispose nursing students to adopt NCS (Ma et al., 2022).

Our study confirmed that MP is significantly positively associated with AP in nursing undergraduates, which is consistent with previous finding (Burns et al., 2000). Many studies have pointed out that the main reasons for individuals to delay tasks that should be completed, knowing that such delay will have adverse consequences are irrational beliefs, such as fear of failure and evaluation anxiety (Beck et al., 2000; Burka and Yuen, 2007). As mentioned in the introduction, maladaptive perfectionists insist on setting unrealistically high standards for themselves, believing that things are either perfect or fail. This requirement makes them often worry that they cannot complete the task, which leads to the fear of failure and indecision (Canter, 2008). These characteristics are consistent with the above-mentioned irrational beliefs that govern procrastination. People who are troubled by these irrational beliefs are more likely to have negative or even catastrophic interpretations of benign events, thereby irrationally delaying many things in life (Steel, 2007). Furthermore, according to the CBT (Knaus, 1973), individual cognition largely determines individual behavior. Therefore, only by helping nursing students develop correct cognitive beliefs can nursing students improve their behavioral performance, including reducing AP.

The results of this study confirmed that resilience was significantly negatively correlated with AP. This is consistent with previous finding (Ko and Chang, 2019). As a positive psychological resource, resilience has a protective effect on an individual’s positive behavior (Richardson, 2002). The important reason why resilience can effectively reduce AP is that it can improve self-esteem and self-efficacy (Richardson, 2002; Warshawski, 2022), enhance psychological resistance to pressure and the temptation of short-term benefits (Rutter, 1999), so that more psychological resources can be devoted to learning, and thus reduce procrastination. Cleary et al. (2018) identified resilience as a necessary trait for nursing students to succeed in learning and practice. Therefore, for nursing educators, improving the resilience of nursing students may be one of the important strategies to reduce AP.

The findings demonstrate that MP is significantly negatively associated with resilience, which is consistent with previous finding (Burns et al., 2000). Maladaptive perfectionists tend to have higher stress levels due to fear of failure and evaluation anxiety. Consequently, there is inevitably a negative impact on resilience (Canter, 2008). In addition, emotions may also be an important reason to explain the impact of MP on resilience. As a new concept in psychology, emotioncy is considered as the blend of emotion and frequency of senses (Miri and Pishghadam, 2021; Pishghadam et al., 2022). It means sense induced emotions can relativize cognition (Pishghadam et al., 2016). According to emotioncy, individuals can be exvolved (hearing and seeing sth) and involved (direct experience of Sth; Miri and Pishghadam, 2021). Individuals with high MP level may lack the sensory experiences of completing the task due to the fear of the task, which will affect their emotioncy level and then have an impact on resilience (Pishghadam et al., 2019). If nursing educators can guide nursing students to lower MP levels and incorporate multiple senses into their educational practice, nursing students tend to experience less stress, which in turn improves resilience.

This study prove that the MP of nursing students not only directly affected AP, but also indirectly affected AP through the partial mediating role of resilience. In the context of nursing education, nursing students are often expected to perform all tasks flawlessly, and they are told that any mistakes can be life-threatening for the patient (Si and Lee, 2022). As a result, nursing students may experience chronic stress due to fear of failure and higher expectations resulting in higher levels of MP (Si and Lee, 2022). Under the long-term effects of this stress, resilience is negatively affected. At the same time, when faced with academic tasks, they are more likely to choose to procrastinate to avoid the execution of tasks. In addition, the moderating effect analysis showed that the resilience of nursing undergraduates with high PCS had a higher predictive effect on AP than that of nursing students with low PCS. This is consistent with the promotion hypothesis of the “protective-protective model” of human development (Cohen et al., 2014). The interaction between the two protective factors enhances each other, and the impact on the outcome variable is a “icing on the cake” effect (Bao et al., 2013). Contrary to our hypothesis, NCS did not moderate the association. One possible explanation is that resilience as a positive coping resource has positive effects on psychology and behavior, whereas NCS is more likely to have negative effects. When the two positive and negative effects coexist, they may cancel each other out, resulting in an insignificant moderating effect of NCS (He et al., 2022).

In current study, on the one hand, we demonstrated ‘how MP works. In other words, MP of nursing undergraduates not only directly affected AP, but also indirectly affected AP through the partial mediating role of resilience. On the other hand, we investigated the when the effect is greater. Namely, the latter half of the mediating path of resilience is moderated by PCS, and the resilience of nursing undergraduates with high PCS has a greater predictive effect on AP. Therefore, the findings of this study will have a certain guiding significance and practical value for nursing educators to improve nursing undergraduates’ academic procrastination.

## Implication for nursing education

The results of this study have important theoretical significance and practical value for improving the academic procrastination of undergraduate nursing students. To reduce the risk of academic procrastination, the following recommendations are made: First of all, for nursing students, it is necessary to develop good study habits and enhance the ability of time management. In the face of heavy learning tasks, nursing students should learn to scientifically disassemble the tasks, break the big tasks into a number of small tasks and complete them one by one. At the same time, through listening to music, physical exercise and other activities to properly adjust the mood, reduce the negative impact of stress. Second, for nursing educators, individual counseling or group counseling can be used to guide nursing students to overcome unreasonable cognitive ways in the process of nursing education practice, so that nursing students do not worry too much about mistakes and reduce the fear of failure. Moreover, from the perspective of positive psychology, nursing educators should pay more attention to the harmonious development of nursing students’ mental health, encourage nursing students to actively cope with the setbacks and challenges in learning and life, so that they can obtain positive emotional experience from solving problems. Finally, for nursing education authorities, psychological experts can be invited to regularly carry out psychological training to help nursing students build a good level of resilience, and share tips to improve their self-control ability, so as to provide a good and warm learning environment for nursing students.

## Limitations

Though there are some highlights, several limitations must be considered. First of all, this study is a cross-sectional study, so further longitudinal studies are needed to investigate the causal association. Secondly, the data used in current research were all self-reported by the participants, which may affect the results by recall bias. Although the deviation of common methods was not found in this study, we can still use a variety of data collection methods (such as the combination of self-report and others’ report) in future studies to ensure the reliability of conclusions. Finally, the participants of this study are only from two undergraduate universities, which hinders the promotion of the conclusion to some extent. Future studies can expand sample sources and explore the differences of results under different cultural backgrounds and educational levels.

## Conclusion

In the context of a global nursing shortage, measures to reduce nursing staff turnover and improve the quality of nursing, such as reducing academic procrastination among nursing undergraduates, are an urgent task. This study found that maladaptive perfectionism, resilience, positive coping style and academic procrastination among nursing undergraduates were significantly correlated between every two variables; resilience partially mediates the association between maladaptive perfectionism and academic procrastination; in the meantime, positive coping style moderated the effect of resilience on academic procrastination. In view of these findings, it is necessary for nursing educators to develop an academic procrastination improvement strategy suitable for nursing undergraduates.

## Data availability statement

The raw data supporting the conclusions of this article will be made available by the authors, without undue reservation.

## Ethics statement

The studies involving human participants were reviewed and approved by Institutional Review Board of Henan Provincial Key Laboratory of Psychology and Behavior (reference: 20220107001). The patients/participants provided their written informed consent to participate in this study. Written informed consent was obtained from the individual(s) for the publication of any potentially identifiable images or data included in this article.

## Author contributions

HH and YD: writing—original draft preparation and investigation. YZ: software and data curation. QP: investigation and validation. YL and XW: supervision and writing—reviewing. CC: conceptualization, methodology, and writing—reviewing and editing. All authors contributed to the article and approved the submitted version.

## Funding

This research was sponsored by Graduate Education Reform and Quality Improvement Project of Henan Province (grant number: YJS2021AL074), Graduate Education Innovation and Quality Improvement Project of Henan University (grant number: SYLYC2022222), and Philosophy and Social Science Planning Research Project of Kaifeng City (grant number: ZXSKGH-2022-1363).

## Conflict of interest

The authors declare that the research was conducted in the absence of any commercial or financial relationships that could be construed as a potential conflict of interest.

## Publisher’s note

All claims expressed in this article are solely those of the authors and do not necessarily represent those of their affiliated organizations, or those of the publisher, the editors and the reviewers. Any product that may be evaluated in this article, or claim that may be made by its manufacturer, is not guaranteed or endorsed by the publisher.
